# Pediatric Thoracic MRI: Safer, Sharper and Smarter Diagnostics

**DOI:** 10.3390/children12111576

**Published:** 2025-11-20

**Authors:** Patricia Tischendorf, Laura Beck, Tobias Krähling

**Affiliations:** Clinic for Radiology, University Hospital Münster, University of Münster, Albert-Schweitzer-Campus 1, Building A1, D-48149 Münster, Germanytobias.kraehling@uni-muenster.de (T.K.)

**Keywords:** thoracic MRI, pediatric, radial imaging, parallel imaging, compressed sensing

## Abstract

Background: Pediatric thoracic magnetic resonance imaging (MRI) has evolved into a valuable diagnostic modality that offers high-resolution morphological and functional assessment. While conventional radiography and computed tomography (CT) remain standard, their radiation exposure poses significant risks in children requiring repeated imaging. Technological innovations have addressed prior MRI limitations such as low lung proton density and motion artifacts, expanding its role in pediatric thoracic imaging. Methods: A review of the recent literature was performed, focusing on technical advancements, key MRI sequences and clinical applications in pediatric thoracic imaging. Emphasis was placed on ultrashort echo time (UTE), phase-resolved functional lung (PREFUL) MRI, hyperpolarized xenon-129 MRI, radial imaging, compressed sensing, parallel imaging and respiratory gating techniques. Results: Modern MRI sequences provide both detailed anatomic visualization and quantitative functional assessment of the pediatric thorax. UTE and PREFUL enable evaluation of lung parenchyma, ventilation, and perfusion, while hyperpolarized gas imaging offers high-resolution functional mapping. Radial, compressed sensing and parallel imaging reduce motion artifacts and acquisition times, enhancing feasibility in uncooperative children. Clinical indications include assessment of congenital malformations, chronic lung disease like cystic fibrosis, infectious and inflammatory disorders, tumors and selected traumatic injuries. Conclusions: Recent technical advances have established pediatric thoracic MRI as a versatile, patient-friendly alternative, as well as a complementary method to CT in selected clinical scenarios. Ongoing developments in acquisition speed, motion compensation and functional imaging are expected to further improve diagnostic accuracy and clinical utility, supporting broader adoption in routine pediatric thoracic evaluation.

## 1. Introduction

Magnetic resonance imaging (MRI) has been firmly established in recent decades as a key diagnostic tool in pediatric thoracic imaging. Both conventional radiography and computed tomography (CT) are widely used due to their rapid availability and high spatial resolution, but the ionizing radiation involved in these methods is particularly concerning in the pediatric age group. For example, in pediatric medicine where repeated imaging may be indicated, having a radiation-free alternative is especially relevant [1]. Early on, MRI was thought not to have a place in lung imaging because of certain physical limitations, particularly low proton density in lung tissue and motion artifacts due to respiration and cardiac motion [2,3]. Nevertheless, the advent of new MRI technologies such as parallel and radial imaging methods, together with the introduction of pioneering specialized sequences like ultrashort echo time (UTE) and phase-resolved functional lung (PREFUL) MRI, has greatly enhanced the feasibility of MRI for pediatric thoracic diagnostics [4,5,6,7,8,9,10,11]. MRI of the thorax provides not only detailed morphologic evaluation of thoracic structures but also functional analysis of the lung. This functional aspect has rendered it a unique and useful tool for screening and monitoring of chronic lung disease conditions like cystic fibrosis, asthma, or interstitial or chronic lung diseases [12,13,14,15]. In addition, MRI is more frequently applied in the assessment of mediastinal masses, pleural diseases and congenital thoracic malformations [16]. Despite technical advancements, thoracic MRI in children is significantly limited by motion artifacts and examination time. However, developments in parallel and radial imaging, as well as respiratory gating techniques, are helping to overcome these drawbacks. Consequently, the potential of MRI as both an alternative and complementary imaging modality to CT in pediatric thoracic imaging is expected to become well established in the future [17]. This article will review the current advances in pediatric thoracic MRI, including routine technical developments, clinical indications and advantages and disadvantages relative to other imaging techniques.

## 2. Challenges of Thoracic MRI

Thoracic MRI is subject to specific technical challenges. The main component of the lung is air cavity with low density of protons. MRI signal is produced by exciting the nuclear spins of hydrogen protons. As the lung is predominantly air, with very little water, the number of measurable protons is thus greatly reduced, resulting in an inherently low signal on traditional MRI scans [2,18]. Moreover, the pronounced magnetic susceptibility differences at the air-tissue interfaces within the lung parenchyma led to local magnetic field inhomogeneities, resulting in rapid signal dephasing, signal loss, and susceptibility-related artifacts. Another major clinical challenge is motion artifacts generated from ongoing respiratory tempo and cardiac rhythm, especially in neonates and infants. Respiration causes periodic displacements of thoracic viscera, and cardiac activity is associated with pulsation-induced deformations. It is widely recognized that motion during image acquisition can significantly degrade image quality, as MRI examinations typically last from several seconds to a few minutes [18]. Therefore, good cooperation and tolerance to undergo the examination are crucial for achieving optimal image quality in pediatric patients. Table 1 summarizes the different imaging modalities of the pediatric thoracic imaging, highlighting their respective advantages and limitations as well as pediatric tolerance in terms of examination duration, acceptance of the examination procedure, and successful examination completion.

## 3. Key Sequences and Techniques

### 3.1. Sequences

**Figure 1:**
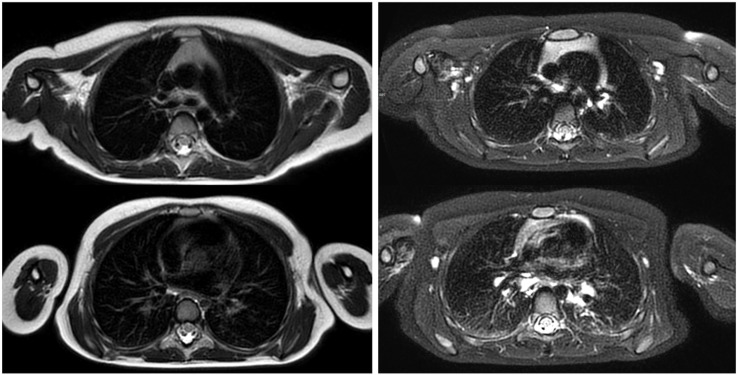
Turbo Spin-Echo sequence of the chest in a 4-year-old girl (**left**) and with spectral inversion and fat saturation (**right**).

Turbo Spin-Echo (TSE) and Fast Spin-Echo (FSE) technique is one of the most commonly used MRI acquisition sequences favored because of its high-quality imaging covering fine detail soft tissue contrast. They provide exquisite depiction of the thoracic anatomy including mediastinum, chest wall, vasculature, and pathologic masses. These characteristics are essential in pediatric thoracic imaging where exact demarcation between congenital anomalies, inflammations, or tumor extensions is frequently mandatory. Another advantage of TSE/FSE is their robustness against patient motion compared to conventional spin-echo techniques, making them suitable for young children who may have difficulty remaining still during scans. This reliability helps to reduce the need for sedation and improves the overall diagnostic yield [3,19,20], pictured in Figure 1.

Gradient-echo (GRE) sequences are characterized by rapid acquisition times, making them highly efficient for capturing dynamic processes in the thorax. They are particularly effective in visualizing vascular structures, cardiac anatomy, and hemodynamics. Since these sequences can display as real-time moving blood passages and cardiac motion, they have a very important role in the assessment of congenital heart disease and monitoring cardiovascular complications arising from pediatric thoracic pathologies. In addition, their wide use in order to combine high temporal resolution and multiphase imaging has given gradient-echo sequences a key role in protocols requiring the assessment of perfusion, valve function or intrathoracic flow dynamics [19,20,21,22,23,24].

Diffusion-Weighted Imaging (DWI) is based on the random motion of water molecules inside biological tissues. By assessing water diffusion properties, DWI enables differentiation between benign and malignant lesions, as well as between acute and chronic changes. Malignant or acute inflammatory lesions typically exhibit restricted diffusion with low apparent diffusion coefficient (ADC) values, whereas benign or chronic fibrotic changes demonstrate higher diffusivity and elevated ADC values. Ischemic, necrotic, or inflamed tissue, often characterized by hypercellular inflammatory infiltrates or reduced extracellular space, also shows restricted diffusion, which can be detected with high sensitivity before alterations on conventional sequences become apparent. Lesions with restricted diffusion, such as malignant or acute inflammatory changes, appear bright on DWI and dark on ADC maps, whereas benign or chronic lesions with facilitated diffusion appear dark on DWI and bright on ADC maps. In the thorax, DWI has emerged as a valuable tool for differentiating benign from malignant lesions, monitoring treatment response, and detecting or excluding inflammatory and infectious processes. Because DWI can be performed without the use of contrast agents, it represents an appealing imaging technique in pediatric patient care, where safety and early detection of disease progression are of particular importance [25,26,27], as shown in Table 2.

**Figure 2:**
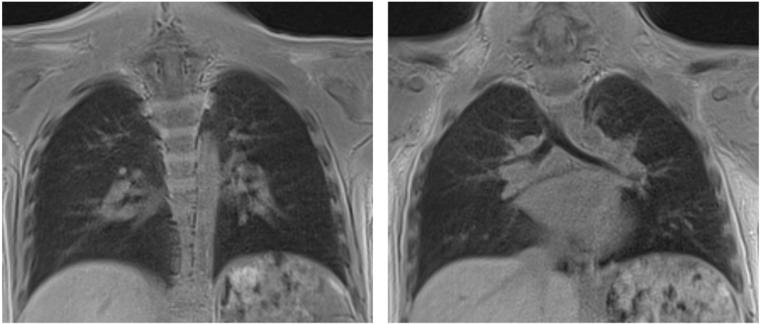
Ultrashort echo time (UTE) MRI sequence of the chest in a 6-year-old boy, demonstrating detailed visualization of the lung parenchyma.

Ultrashort Echo Time (UTE) sequences represent a breakthrough in pediatric thoracic MRI. Conventional sequences often fail to capture signals from tissues with extremely short T2* relaxation times, such as lung parenchyma. UTE overcomes this limitation by acquiring data within microseconds, thereby enabling visualization of pulmonary structures previously considered “MRI-invisible.” This capability has proven transformative for pediatric patients with chronic or congenital lung diseases such as cystic fibrosis, bronchopulmonary dysplasia or interstitial lung disease. Beyond improving structural assessment, UTE also opens the door to functional applications and may reduce reliance on CT and its associated radiation exposure [7,11,28] (Figure 2).

### 3.2. Techniques

**Figure 3:**
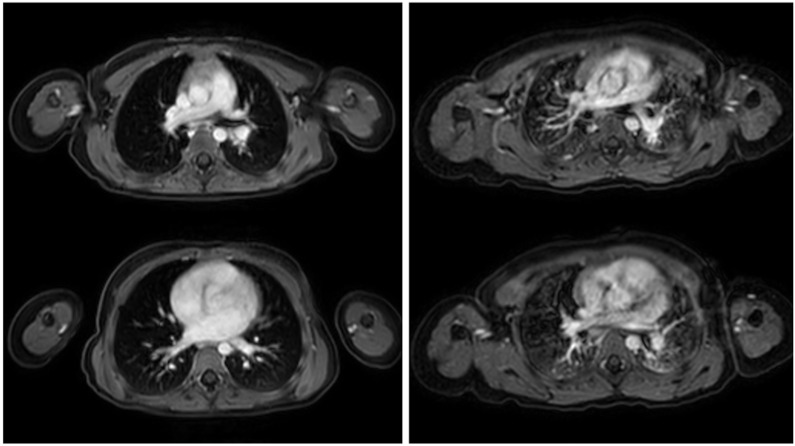
Side-by-side comparison of a post-contrast T1-weighted sequence acquired with a navigator-based technique (**left**) and a post-contrast T1-weighted sequence obtained during breath-hold (**right**), demonstrating respiratory motion artifacts in the latter.

Respiratory motion is a major challenge in thoracic MRI, particularly in pediatric patients who are unable to perform reliable breath-holds. To mitigate respiratory motion artifacts, respiratory gating and navigator-based triggering techniques have been developed. Respiratory gating relies on external sensors, such as respiratory bellows, to monitor the breathing cycle and trigger image acquisition during a specific phase, typically end-expiration, when motion is minimal. Navigator-based triggering, in contrast, uses MR signals to track diaphragmatic motion directly by acquiring a small navigator echo that monitors diaphragm position and allows data acquisition only within a defined acceptance window. These motion-compensated approaches synchronize image acquisition with the respiratory cycle, substantially reducing motion artifacts and improving anatomical sharpness. In pediatric imaging, they enable the acquisition of high-quality diagnostic images during free breathing, allowing accurate assessment of pulmonary and mediastinal structures without compromising patient comfort or safety, despite prolonged acquisition times for individual sequences [29,30,31,32], as seen in Figure 3.

Parallel imaging is an advanced MRI technique that accelerates data acquisition by leveraging the spatial sensitivity profiles of phased-array coils. In this approach, k-space is undersampled by skipping measurement of specific k-space lines to shorten scan time while maintaining spatial resolution. The resulting aliasing artifacts are corrected through specialized reconstruction algorithms that utilize spatial coil sensitivity information [33,34]. Unlike conventional imaging, where signals from multiple coils are combined prior to reconstruction, parallel imaging processes and amplifies each coil signal independently through separate receiver channels [35].

This technique enables substantial scan time reduction or improved spatial resolution and is particularly beneficial in motion-prone regions such as the abdomen, heart, and vasculature. Major limitations include a reduced signal-to-noise ratio (SNR) and possible residual aliasing artifacts; however, the SNR loss can be partially mitigated by imaging at higher field strengths (e.g., 3 T) [36]. In pediatric MRI, parallel imaging minimizes motion-related artifacts and facilitates high-resolution assessment of thoracic anatomy and pathology [37,38].

Compressed sensing is another MRI acceleration technique that relies on undersampling of k-space data; however, the undersampling is performed in a pseudo-random pattern, producing incoherent, noise-like artifacts rather than the structured ghosting or aliasing typically seen with parallel imaging methods. Beyond pseudo-random k-space undersampling, compressed sensing exploits the intrinsic sparsity of MR images and employs iterative, nonlinear reconstruction algorithms to recover missing data [39]. The acquired data are decompressed, filtered, and iteratively reconstructed to produce the final image, effectively reducing noise and preserving image details. In contrast to parallel imaging, compressed sensing reconstruction is independent of coil geometry. The technique is compatible with other acceleration approaches, including parallel and radial imaging, and has demonstrated robust feasibility at high-field MRI systems [40]. At lower magnetic field strengths; however, the reduced SNR may limit its performance [35]. Compressed sensing has significantly advanced imaging sequences that are sensitive to respiratory motion, achieving accelerated acquisition while maintaining high spatial and temporal resolution. It has shown particular efficacy in free-breathing, radially acquired, contrast-enhanced 3D gradient-echo MRI used for multiphasic contrast-enhanced imaging [41,42]. In pediatric MRI, it has proven to be a reliable and robust technique for free-breathing contrast-enhanced MR angiography and cardiac cine imaging, where motion and limited patient cooperation often pose challenges [43].

**Figure 4:**
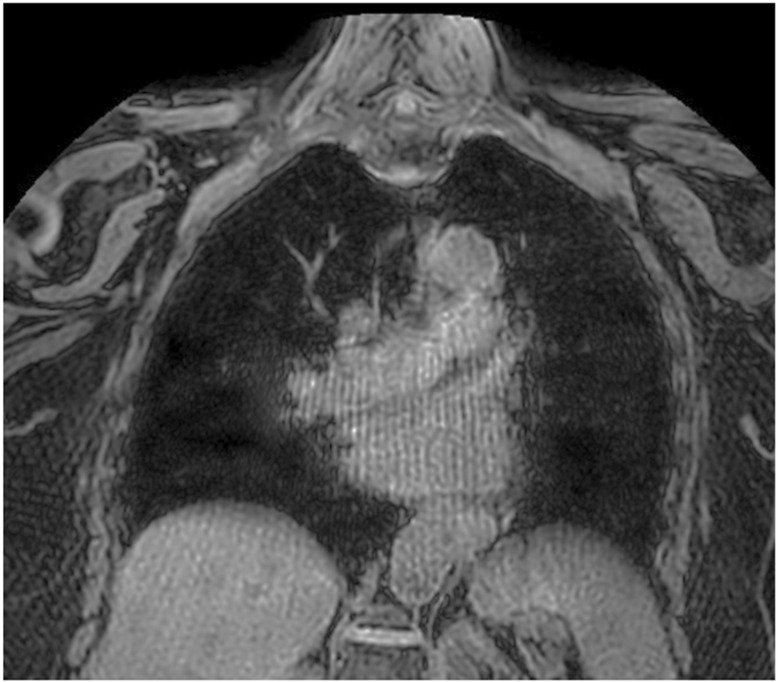
Unenhanced radial T1-weighted MRI sequence showing streak artifacts in the coronal plane.

In radial imaging, k-space is sampled along rotating lines that all cross through the center of the k-space like the spokes of a wheel, rather than along parallel lines of a Cartesian readout [39]. Radial readout can not only be achieved by individual lines alone, but also by small groups of parallel lines that from rotating ‘blades’. A major advantage of this technique is its reduced sensitivity to motion. Unlike Cartesian sampling, where motion in the phase-encoding direction produces coherent and easily visible artifacts, motion-related inconsistencies in radial imaging are dispersed across multiple directions, making them less noticeable. Since every radial line passes through or near the center of k-space, motion affects only a small amount of data, thereby preserving the quality of the overall image [35]. Furthermore, because the center of k-space is oversampled in radial acquisition, the SNR is preserved [44]. However, radial imaging is associated with several potential drawbacks, including streak artifacts, blurring of anatomical boundaries due to angular undersampling of the k-space periphery, the need for multiple acquisitions to reduce undersampling effects, and increased sensitivity to magnetic field inhomogeneities (Figure 4). When undersampling occurs, aliasing manifests as streaking artifacts rather than overlapping artifacts, as typically seen in undersampled Cartesian acquisitions.

Radial k-space sampling techniques can be applied to both FSE and GRE sequences and are also compatible with several other acceleration techniques. This technique is particularly advantageous for free-breathing acquisitions and has a significant advantage over traditional Cartesian imaging due to lesser motion artifacts, especially in pediatric thoracic MRI [32]. Additionally, the radial symmetric nature of the data acquisition makes it more robust towards any distortion caused by breathing and cardiac motion. The second main benefit is that it enhances the visualization of the air-tissue interface. Standard MRI suffers from strong susceptibility effects at the interfaces between air-laden lung structures and surrounding soft tissue, resulting in image distortions. By continuously acquiring data at the center of k-space, radial imaging reduces artifacts, improves the visualization of pulmonary anatomy, and provides a more uniform signal distribution with increased perceived image sharpness [37,45]. Thus, offering better characterization of the lung parenchyma and mediastinal structures, which is important in the detection and follow-up of thoracic pathologies [46]. In addition, due to its insensitivity to motion artifacts, it is particularly suited for children, who are unable to remain still, thereby reducing the need for general anesthesia during MRI examinations [47,48]. This makes it a promising modality in pediatric imaging as it provides a greater accuracy and patient-friendly alternative to standard MRI techniques (Table 3)

### 3.3. Experimental Techniques

Phase-Resolved Functional Lung (PREFUL) MRI provides a unique approach for evaluating pulmonary function without the need for contrast agents or breath-holding maneuvers. By analyzing cyclic signal fluctuations arising from normal respiration and cardiac activity, PREFUL generates maps of regional ventilation and perfusion. In these images, color-coded representations of ventilation and perfusion are visualized, with regions of reduced airflow or blood flow appearing as areas of lower signal intensity or altered color. This technique is particularly advantageous in pediatrics, where patient cooperation with breath-hold imaging is often limited. The ability of PREFUL to non-invasively and child-friendly assess lung function makes it a promising tool for diagnosing and monitoring obstructive and restrictive lung diseases in children [8,10,49]. Pöhler et al. [51] further demonstrated that PREFUL MRI can identify a distinct pulmonary perfusion phenotype characterized by reduced regional blood flow in children and adolescents with post-COVID-19 condition.

Hyperpolarized Xenon-129 MRI marks a new milestone in pulmonary imaging. Following inhalation of hyperpolarized gas, patients achieve markedly enhanced signal intensity, allowing for high-resolution visualization of ventilation patterns, gas distribution, and microstructural alterations of the lung. In addition to structural imaging, this technique provides insights into alveolar gas exchange and perfusion. In hyperpolarized xenon-129 MRI, the lungs appear bright in well-ventilated regions and dark in areas of airflow limitation. Color-coded maps often highlight ventilation and gas exchange, allowing visualization of regional defects and impaired alveolar function with high spatial detail. The potential of Xenon-129 MRI in pediatrics lies in its ability to detect early, subtle lung injury that may be missed with conventional imaging. This enables earlier intervention and longitudinal monitoring of diseases such as asthma, cystic fibrosis, or post-infectious lung damage [17,50].

## 4. Clinical Indications for Pediatric Thoracic MRI

MRI is of paramount importance in several clinical contexts and is becoming an essential tool in the evaluation of thoracic disease in children. An example MRI protocol for pediatric patients aged approximately 3–6 years is summarized in Supplementary Table S1, including detailed sequence parameters obtained on a Philips 1.5 T system (Philips Healthcare, Best, The Netherlands).

Assessment of congenital anomalies is one of the important applications of MRI. Imaging plays an important role in identifying various congenital malformations such as lung malformations, vascular malformations and bronchopulmonary abnormalities. It also helps to differentiate other congenital pulmonary entities such as bronchopulmonary sequestration and congenital cystic adenomatoid malformations, the clinical management of which differs significantly [52,53,54].

In cystic fibrosis and other obstructive lung diseases, MRI has the potential major advantage of allowing functional characterization of lung. This is especially critical for long-term disease tracking. In addition, techniques such as diffusion-weighted imaging are helpful for the identification of inflammatory processes and obstructive changes that otherwise would not be detected [14,55,56,57,58].

**Figure 5:**
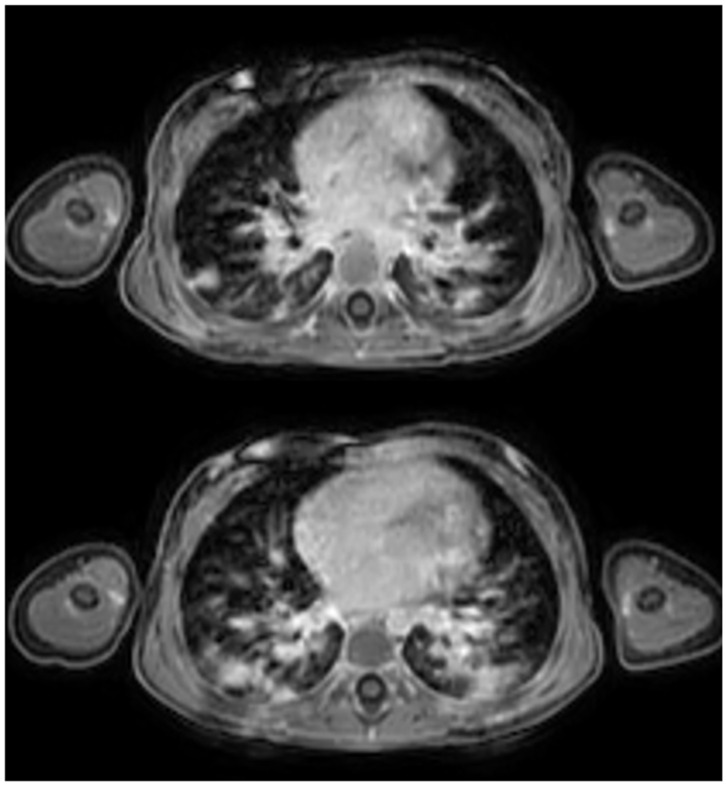
Post-contrast gradient-echo T1-weighted sequence of the chest in a 5-year-old boy, showing pneumonic consolidations in both lower lobes.

As shown in Figure 5, MRI is also beneficial in the evaluation of infectious diseases for children suffering from recurrent pneumonias or tuberculosis. By providing detailed diagnostic information, MRI helps distinguish between acute and chronic infections. Its high sensitivity in detecting pleural effusions, lung abscesses and mediastinal infections makes it a powerful tool for comprehensive disease assessment [59,60,61,62].

For pulmonary tumors, MRI excels in differentiating between benign and malignant neoplasms and accurately assessing the extent of tumor spread. Its superior soft tissue contrast allows for precise localization and classification of tumors. This is particularly advantageous for neurogenic tumors in the posterior mediastinum, where MRI surpasses CT in visualizing potential spinal cord involvement, enabling better treatment planning and surgical decision-making [15,23].

In interstitial lung diseases, MRI serves as a diagnostic tool for disease monitoring [63,64,65]. CT establishes the gold standard for the identification of subtle interstitial alterations, while MRI, with novel methods such as UTE and PREFUL, opens a window for unique and important functional lung evaluation. These findings enable the evaluation of disease progression and aid in the optimization of personalized treatment approaches.

MRI is also crucial for monitoring immunological and rheumatological diseases. Chronic carditis, interstitial fibrosis, pulmonary hypertension, obstructive lung disease and pleural effusion can be seen in children with systemic lupus erythematosus or juvenile idiopathic arthritis. MRI sensitivity is notably increased for early inflammatory changes in the lung parenchyma and pleural structures by utilizing diffusion-weighted and contrast-enhanced images. Such an approach allows for the recognition of even subclinical lung involvement, facilitating prompt intervention and disease management [66].

Although CT is commonly the diagnostic modality of choice for traumatic injuries, MRI can serve as a useful adjunct in selected cases, particularly for follow-up imaging. It is exceptionally sensitive when it comes to detecting soft-tissue injuries, hematomas, and parenchymal damage [67].

## 5. Discussion

Recent developments show that pediatric thoracic magnetic resonance imaging (MRI) has progressed from an experimental technique to a clinically useful diagnostic tool. When compared with conventional radiography and computed tomography (CT), MRI has the essential advantage of being free of ionizing radiation and is thus especially well-suited for the pediatric population that may need multiple follow-up examinations [15,56]. Aside from detailed morphologic assessment, MRI is unique in that it can provide functional information about ventilation and perfusion, a feature CT is only able to cover to some extent [68]. Technological innovations such as ultrashort echo time (UTE) [11], phase-resolved functional lung (PREFUL) MRI [49] and hyperpolarized xenon-129 MRI [50] have overcome fundamental limitations of lung MRI. The capacity to image parenchymal structures with very short T2* relaxation times while simultaneously acquiring a functional component and the lack of the use of contrast media or breath holds are important advances. Moreover, methodologies such as radial imaging, parallel imaging and respiratory gating substantially decrease motion artifacts and enhance image quality for free breathing scans, which makes it more feasible in non-cooperative pediatric patients [17] and reduces the need for general anesthesia [48]. However, general anesthesia or sedation may still be required in very young children and infants to ensure adequate image quality.

However, there are still large obstacles to clinical applicability. Available MRI systems and dedicated sequences are limited to the field of tertiary care or research institutions. MRI examinations take longer and require more protocols than CT, which remains the preferred modality in acute emergency cases due to its speed, availability, and standardized procedures. In addition, CT remains more sensitive to very small lesions and subtle interstitial changes that remain a limitation of MRI despite ongoing advancements. Faced with the highly complex pediatric chest, MRI is also another challenge: the paucity of standardized cutoff criteria and protocols. Advanced workflows have been developed at the individual center level, but there is still no multicenter harmonization. This is especially true for the longitudinal diagnosis of chronic diseases like cystic fibrosis or interstitial lung disease, where quantitative and consistent imaging biomarkers would be of great importance in patient care. There are a number of promising developments. Reconstruction and motion correction using artificial intelligence may reduce acquisition times with additional enhancements in image quality. The judicious application of functional MRI in the clinical setting is likely to improve its diagnostic and prognostic utility.

In conclusion, pediatric thoracic magnetic resonance imaging offers a radiation-free method complementary to computed tomography and radiography, which can be used for combined anatomic and functional evaluation. Recent developments have led to enhanced image quality while reducing examination time. Once more widely available and supported by standardized protocols, MRI will become an essential tool for the diagnosis, follow-up, and therapy planning of pediatric thoracic diseases.

## Figures and Tables

**Table 1:** Comparison of Imaging Modalities in Pediatric Thoracic Assessment.

Imaging Modality	Radiation Exposure	Morphological Resolution	Functional Assessment	Availability	Pediatric Tolerance
Conventional radiography	Yes (low)	Low	No	High	High
Computed tomography	Yes (high)	Very High	Limited (indirect)	High	Moderate
Magnetic resonance imaging	No	Moderate–High	Possible	Moderate	Moderate
Ultrasound	No	Low (superficial only)	Limited	High	High

**Table 2:** MRI Sequences for Pediatric Thoracic Imaging. Turbo Spin-Echo (TSE); Fast Spin-Echo (FSE); Gradient-echo (GRE); Diffusion-Weighted Imaging (DWI); Ultrashort echo time (UTE).

Sequence	Primary Utility	Advantages	Pediatric Relevance	Reference
TSE/FSE	Anatomic detail of mediastinum & chest wall	Excellent soft tissue contrast	Clear visualization of mediastinum, chest wall	[3,19,20]
GRE	Dynamic imaging	Fast acquisition, cardiac imaging	Functional cardiovascular evaluation	[19,20]
DWI	Inflammatory/tumor detection	Microstructural imaging without contrast agent	Useful for infection and tumor staging	[25,26,27]
UTE	Parenchymal lung imaging	Visualizes previously “invisible” lung structures	Detect structural lung changes	[7,11,28]

**Table 3:** MRI Techniques for Pediatric Thoracis Imaging.

Technique	Primary Utility	Advantages	Pediatric Relevance	References
Respiratory Gating/Navigator	Motion reduction	Sharper images	Reduced need for general anesthesia	[29,30,31,32]
Parallel Imaging	Faster acquisition	Less motion artifact	Reduced need for general anesthesia or sedation	[37,38]
Radial Imaging	Artifact suppression	Robust to motion, improved air–tissue interface visualization	Suitable for restless or sedated patients	[47,48]
Phase-Resolved Functional Lung	Perfusion & ventilation imaging	Free-breathing, functional imaging	Ideal for young/uncooperative children	[8,10,49]
Hyperpolarized Xenon-129	Ventilation/perfusion & gas exchange	Exceptional functional assessment	Non-invasive functional lung assessment	[17,50]

## Data Availability

Data sharing is not applicable.

## Supplementary Materials

The following supporting information can be downloaded at: https://www.mdpi.com/article/10.3390/children12111576/s1, Table S1: An example MRI protocol for pediatric patients aged ~3–6 years is summarized, including detailed sequence parameters obtained on a Philips 1.5 T system.

## Abbreviations

CT	Computed Tomography
DWI	Diffusion-Weighted Imaging
FSE	Fast Spin-Echo
GRE	Gradient-Echo
MRI	Magnetic Resonance Imaging
PREFUL	Phase-Resolved Functional Lung
SNR	Signal-to-Noise Ratio
TSE	Turbo Spin-Echo
UTE	Ultrashort Echo Time
