# Supplementary material for: Pediatric Thoracic MRI: Safer, Sharper and Smarter Diagnostics
Source: Children (Basel). 2025 Nov 20;12(11):1576. doi: 10.3390/children12111576 (PMC12651427; doi:10.3390/children12111576)
Supplement: Supplementary file 1 [file children-12-01576-s001.zip › children-3951404-supplementary.pdf]

	T2 SPIR MVXD coronal	T2 MVXD axial	3D T1w VANE mDIXON axial	T2 MVXD SPAIR axial GD	3D T1w VANE mDIXON axial GD	DWIBS
Technique	Multishot radial blades TSE	Multishot radial blades TSE	Singleshot radial TFE	Multishot radial blades TSE	Singleshot radial TFE	Singleshot IR-EPI
Acq voxel size	1.31 x 1.31	1 x 1	1.35 x 1.35	1 x 1	1.35 x 1.35	2.21 x 2.25
Recon voxel size (mm)	0.68 x 0.68	0.78 x 0.78	0.78 x 0.78	0.78 x 0.78	0.78 x 0.78	0.77 x 0.77
Field of view (mm)	350 x 350x 112	300 x 300 x 145	300 x 300 x178	300 x 300 x 145	300 x 300 x 178	310 x 310 x 245
Repetition time (ms)	2276	3079	4.9	5062	4.9	4858
Echo time (ms)	80	100	1.49/ 2.6	100	1.49/ 2.6	94
Flip angle (°)	90	90	10	90	10	na
Number of averages	2	1	1	1	1	6
Slice thickness (mm)	3	3	3	3	3	4
Spacing between slices (mm)	3.3	3.3	- 1.5	3.3	- 1.5	0
Respiratory triggering	bellow	bellow	navigator	bellow	navigator	bellow
TSE Factor	35	39	na	39	na	na
Sense	yes (1.4)	yes (1.4)	no	yes (1.4)	no	yes (2.0)
B- values	na	na	na	na	na	0, 1000

Table S1: An example MRI protocol for pediatric patients aged ~3- 6 years is summarized, including detailed sequence parameters obtained on a Philips 1.5 T system; DWIBS= Diffusion- Weighted Imaging with Background Suppression; GD= Gadolinium; IR- EPI= Inversion Recovery Echo Planar Imaging; MVXD= MultiVane extended; SPAIR= Spectral Adiabatic Inversion Recovery; SPIR = Spectral Presaturation with Inversion Recovery; TFE= Turbo Field- Echo; TSE= Turbo Spin- Echo; VANE= Variable Number of Excitations.
